# Supplementary material for: Controlling Calcium Carbonate Particle Morphology, Size, and Molecular Order Using Silicate
Source: Materials (Basel). 2021 Jun 24;14(13):3525. doi: 10.3390/ma14133525 (PMC8269534; doi:10.3390/ma14133525)
Supplement: Supplementary file 1 [file materials-14-03525-s001.zip › materials-1236953-supplementary.pdf]

Supplementary material

Silicon as a key element in controlling calcium carbonate particle morphology, size and molecular order

Lior Minkowicz[1], Arie Dagan[1], Vladimir Uvarov[2], Ofra Benny[1]

1. The Institute for Drug Research, The School of Pharmacy, Faculty of Medicine, The Hebrew University, Ein-Kerem Campus, Jerusalem, 91120, Israel
2. The Hebrew University of Jerusalem, The Faculty of Natural Sciences, The Center for Nanoscience and Nanotechnology, The Unit for Nanoscopic Characterization, E. Safra Campus, Givat Ram, Jerusalem, 91904, Israel

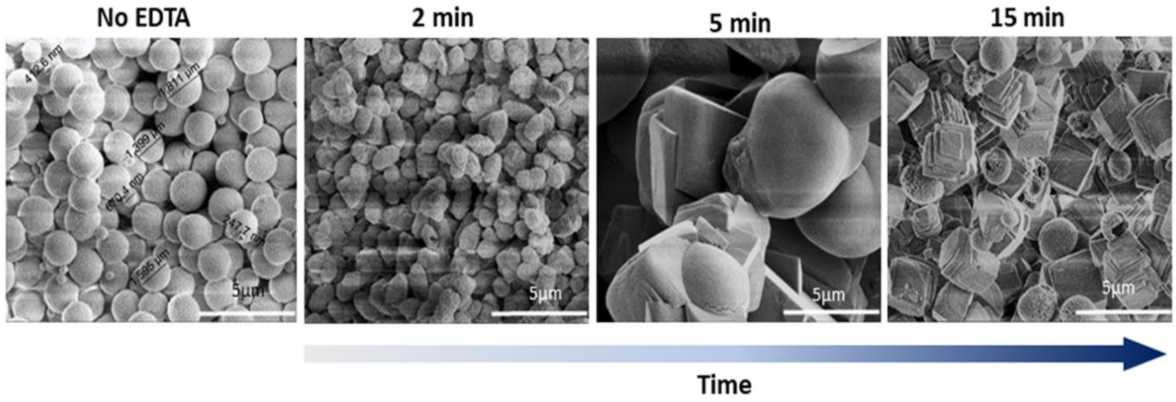

Figure S1: Effect of EDTA addition on the calcium carbonate end product. EDTA, 50 µl of 0.25 M was added and stirred for designated periods of time, 2, 5 and 15 minutes. Representative images of dry samples were taken by SEM. The morphology of the precipitated particles is shown with and without EDTA addition. Samples without EDTA were stirred for 2 minutes resulting in heterogenic samples containing various particle shapes. Bar =5 micron.

Entire sample

Element	App Conc.	Intensity Corrn.	Weight%	Weight% Sigma	Atomic%
C K	9.66	1.4088	12.95	0.25	22.06
O K	24.76	1.0036	46.60	0.27	59.60
Si K	0.46	1.0960	0.79	0.05	0.58
Ca K	17.95	1.0103	33.56	0.23	17.13
Au M	2.17	0.6721	6.09	0.20	0.63
Totals			100.00		

Sphere

Element	App Conc.	Intensity Corrn.	Weight%	Weight% Sigma	Atomic%
C K	4.95	1.3780	4.90	0.18	10.16
O K	23.32	0.8541	37.16	0.25	57.88
Si K	0.64	1.1324	0.77	0.05	0.68
Ca K	37.39	1.0486	48.56	0.25	30.19
Au M	4.40	0.6950	8.62	0.20	1.09
Totals			100.00		

Irregular

Element	App Conc.	Intensity Corrn.	Weight%	Weight% Sigma	Atomic%
C K	2.87	1.4399	4.74	0.21	11.05
O K	7.74	0.7206	25.59	0.32	44.73
Ca K	27.72	1.0689	61.79	0.36	43.11
Au M	2.34	0.7075	7.87	0.27	1.12
Totals			100.00		

Figure S2: Silicon element found in sphere particles and not in irregular shaped-particles following EDS elemental analysis of CaCO3. EDS chemical elemental analysis of a selected sample containing microspheres and particles with other shapes. The analyses are of the overall sample, single spheres and single irregular shaped particles. Elemental analysis of the entire sample as well as the specifically shaped particles is provided. Small amounts of silicon atoms were found in the entire sample analysis and in the spherical particles' analysis, while no silicon was detected in non-spherical particles. Gold element (Au) detected is due to sample coating prior to SEM imaging.

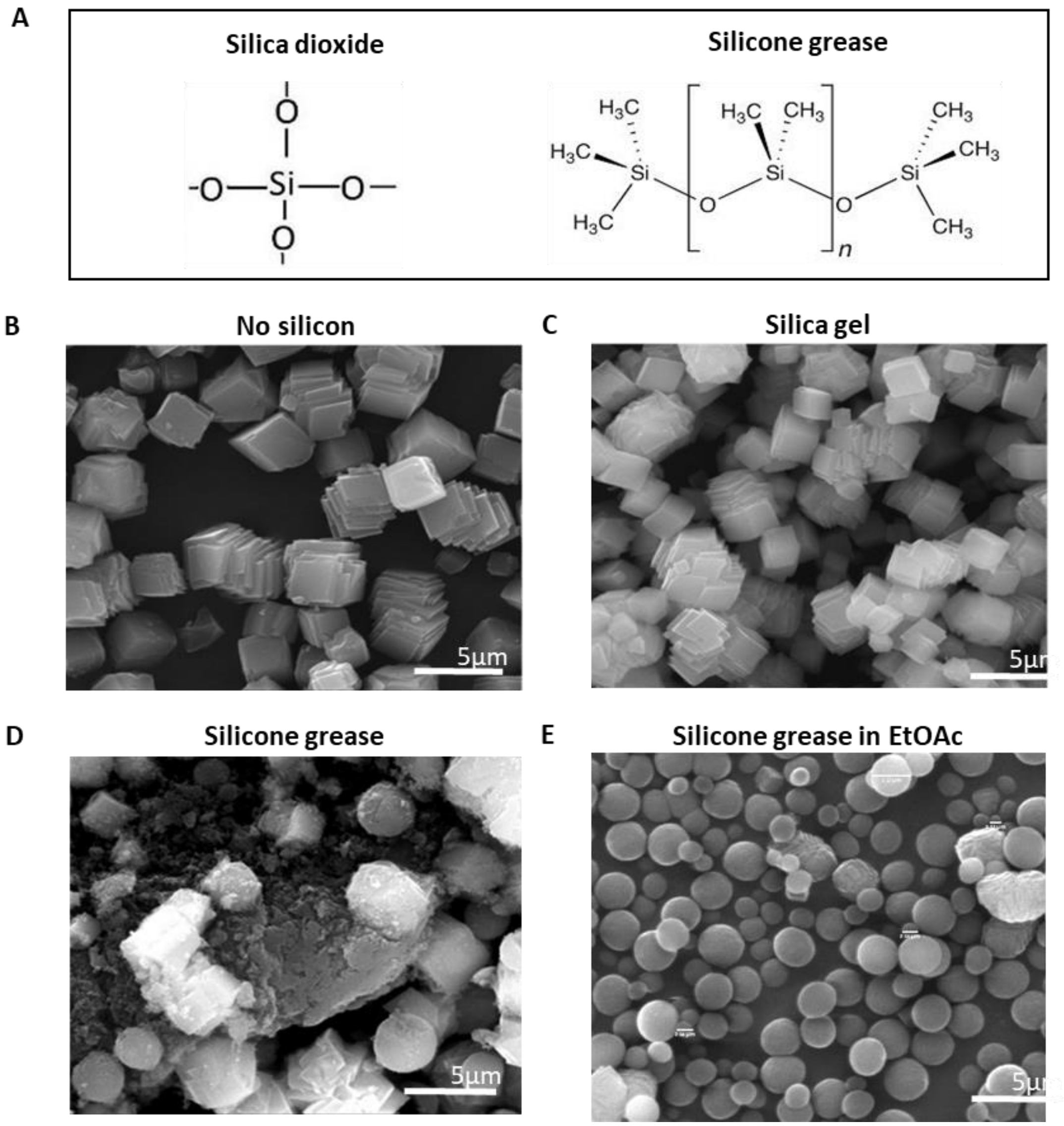

Figure S3: Effect of silicon containing additives - silica gel and silicone grease in the fabrication of particles. A) Chemical structures of the mix additives used to fabricate the particles. **B)** Control particles fabricated without any additives. **C)** Particles fabricated with excess of silica gel (Silica dioxide). **D)** Particles fabricated with excess of silicone grease. **E)** Silicone grease was dissolved in EtOAc (5% w/v) and used 0.1 ml with CMC in the synthesis reaction. Particles fabricated this way had a smaller diameter and were mostly spherical. Bar= 5 micron.

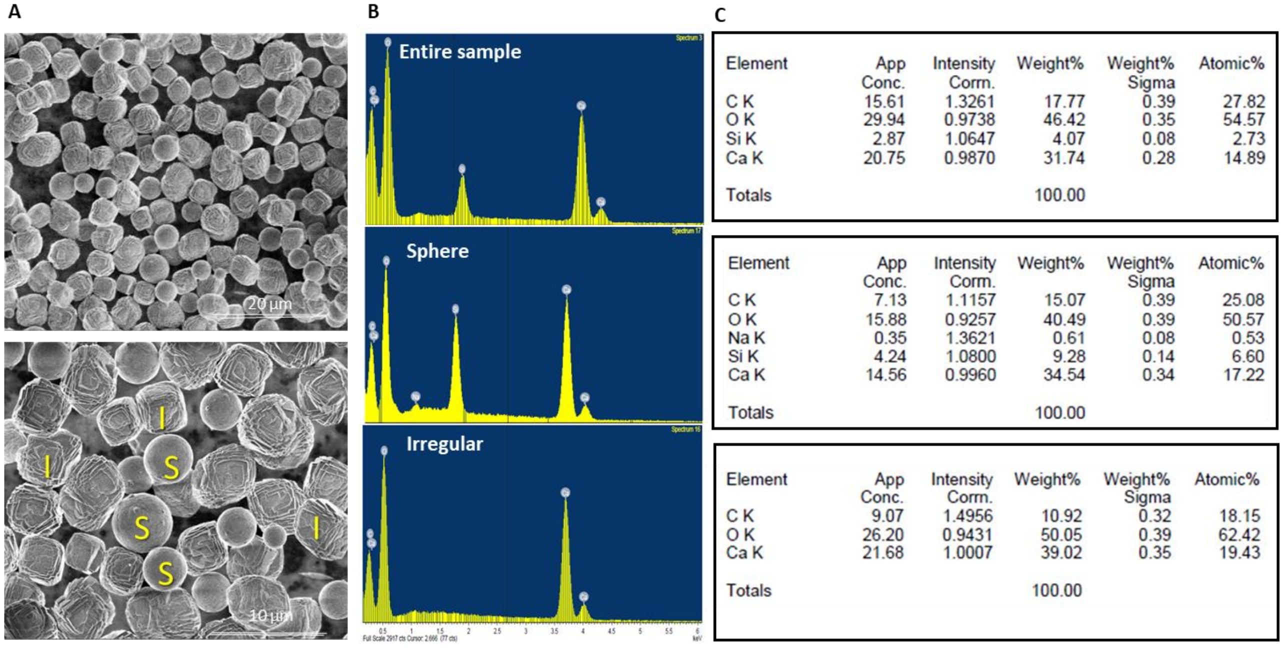

C

Element	App Conc.	Intensity Corrn.	Weight%	Weight% Sigma	Atomic%
C K	15.61	1.3261	17.77	0.39	27.82
O K	29.94	0.9738	46.42	0.35	54.57
Si K	2.87	1.0647	4.07	0.08	2.73
Ca K	20.75	0.9870	31.74	0.28	14.89
Totals			100.00		

Element	App Conc.	Intensity Corrn.	Weight%	Weight% Sigma	Atomic%
C K	7.13	1.1157	15.07	0.39	25.08
O K	15.88	0.9257	40.49	0.39	50.57
Na K	0.35	1.3621	0.61	0.08	0.53
Si K	4.24	1.0800	9.28	0.14	6.60
Ca K	14.56	0.9960	34.54	0.34	17.22
Totals			100.00		

Element	App Conc.	Intensity Corrn.	Weight%	Weight% Sigma	Atomic%
C K	9.07	1.4956	10.92	0.32	18.15
O K	26.20	0.9431	50.05	0.39	62.42
Ca K	21.68	1.0007	39.02	0.35	19.43
Totals			100.00		

Figure S4: SEM and EDS analysis of particles fabricated with silicone grease and CMC. A sample of fabricated particles obtained with the same procedure using excess of silicone grease and 25 mg of CMC. **A)** SEM images of the particles that were analyzed in EDS. Cubes and spheres are present the sample. **B), C)** EDS analysis of the entire sample, the spheres and the irregular shaped particles. Silicon was found when analyzing the entire sample or individual sphere particles, while no silicone was found in the irregular shapes. Sodium (Na) traces found are due to the sodium carbonate reagent.

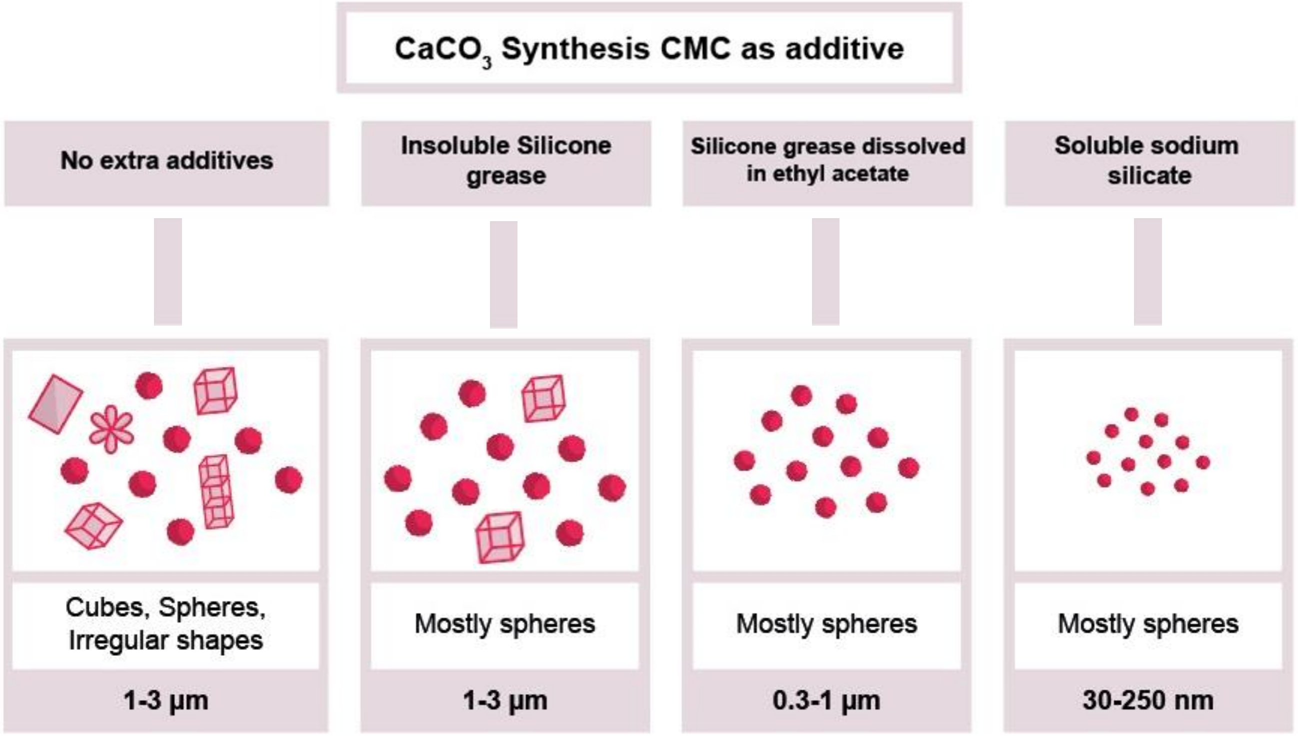

Figure S5: Summary of the different particle types observed using different silicon containing additives. Calcium carbonate synthesized without additives resulted in various morphological shapes, size ranges of 1-3 microns. Addition of silicone grease to the synthesis environment resulted in particles of the same size range but with a larger fraction of spherical shapes. Dissolving the silicone grease in EtOAc resulted in particles of a smaller diameter, 0.3-1 microns. Synthesis with sodium silicate reduced the particles' size further to 30-250 nanometers.
